# Bladder cancer-induced CVD mortality: Role of CAPG protein

**DOI:** 10.1371/journal.pone.0338101

**Published:** 2026-02-17

**Authors:** Junwen Shen, Zhucheng Zhao, Zhaojun Li, Rongjiang Wang

**Affiliations:** 1 The Department of Urology, The First Affiliated Hospital of Huzhou Normal College, Huzhou, Zhejiang, China; 2 Huzhou Key Laboratory of Precise Diagnosis and Treatment of Urinary Tumors, Huzhou, Zhejiang, China; Universita degli Studi della Campania Luigi Vanvitelli, ITALY

## Abstract

**Objective:**

Explore the causes and mechanisms of bladder cancer-induced Cardiovascular diseases (CVD) death.

**Method:**

We acquired bladder cancer patient data from the SEER database to evaluate CVD death risk. Cross-WGCNA was employed to identify comorbidity genes linking bladder cancer and heart failure. Functional phenotypes of bladder cancer cell lines were analyzed using cell culture, transaction, CCK-8, and Transwell assays, while ELISA determined extracellular target protein concentrations. Myocardial cell function was assessed by examining cell proliferation, collagen I levels, and mitochondrial membrane potential.

**Result:**

Our study, analyzing 140,760 bladder cancer patients from the SEER database, revealed that CVD is a major cause of death, increasing risk by 18%. Cross-WGCNA and Lasso regression identified SFRP1 and CAPG as key serum proteins linked to bladder cancer and heart failure. Regulating these proteins’ mRNA levels significantly impacts cancer proliferation, migration, and invasion. CAPG, in particular, suppresses myocardial cell function. We discovered SB525334 as a strong CAPG inhibitor in bladder cancer cells, potentially enhancing cisplatin’s effectiveness by targeting CAPG.

**Conclusion:**

Bladder cancer patients face an elevated CVD death risk due to high CAPG protein expression, which can raise serum CAPG levels and harm cardiomyocytes.

## Background

Bladder cancer is a common malignancy of the urinary system, with increasing incidence and mortality worldwide [1]. Despite continuous improvements in treatment methods for bladder cancer, patients still face many challenges in terms of prognosis. When considering the causes of death among bladder cancer patients, the primary focus is on mortality resulting from the primary disease. However, numerous studies have reported an increased risk of death from other diseases among bladder cancer patients, with Cardiovascular diseases death (CVD) emerging as a significant cause of mortality beyond the primary bladder cancer [2].

Why do patients with bladder cancer induce CVD? This is an academic question that remains to be further explored. Currently, the specific connection between bladder cancer and CVD, as well as its underlying mechanisms, are not fully understood [3]. Firstly, the anatomical distance between the bladder and the heart, coupled with their distinct physiological functions, has traditionally made it difficult to directly associate bladder cancer with heart disease. Secondly, with the advancement of systems biology and molecular medicine, there is growing evidence that malignant tumors can affect the function of distant organs, including the heart, through various pathways [4].

One potential mechanism is that bladder cancer may influence heart function by secreting specific serum proteins. These serum proteins, which may include cytokines, growth factors, hormone-like substances, and more, can enter the bloodstream and bind to receptors on heart cells, thereby regulating the growth, differentiation, and metabolism of these cells. In patients with bladder cancer, alterations in the levels of these serum proteins may adversely affect the heart, increasing the risk of CVD [5].

Through this study, we have identified a key serum protein, CAPG. Bladder cancer increases the concentration of serum protein CAPG, which inhibits the proliferation of cardiomyocytes and membrane potential. Additionally, a small molecule drug (SB525334) was found which enhanced the therapeutic effect of cisplatin on bladder cancer and suppressed the level of CAPG protein in bladder cancer.

## Methods

### Acquisition of bladder cancer patient data from the SEER database

This study relied on data from the SEER (Surveillance, Epidemiology, and End Results) database, specifically the version released in November 2020 [6]. The pertinent patient population was identified and retrieved using SEER*Stat Software Version 8.3.9.2, which incorporates population-based data from bladder cancer registries. Comprehensive bladder cancer patient records, including chemotherapy and radiotherapy treatments, were extracted for the time frame spanning January 1, 2000, to December 31, 2017. Detailed patient information was gathered, encompassing factors such as age at diagnosis, racial background (categorized as white, black, other, or unknown), marital status (including married, divorced, separated, single, widowed, unmarried, domestic partner, or unknown designations), disease stage (classified as I, II, III, or IV), TNM staging (ranging from T1-T4, N0-N3, and M0-M1), surgical procedures undergone, and chemotherapy and radiotherapy treatment histories.

### Risk of bladder cancer associated CVD (Cardiovascular diseases) death

In this study, we conducted a comparative analysis of all-cause and cardiovascular disease (CVD) mortality rates among bladder cancer patients relative to the general US population. To quantify this, we employed the standardized mortality rate (SMR), which was calculated by dividing the observed deaths in the bladder cancer patient population by the expected deaths based on data from relevant research. The expected deaths for the US population were derived from pertinent research findings [7]. Furthermore, we computed 95% confidence intervals (CIs) for the SMR to assess the potential risk of CVD associated with the tumor.

### The data collection

After conducting a comprehensive search for datasets pertaining to various CVDs (cardiovascular diseases), it was determined that only heart failure had both mRNA expression and serum protein datasets available for public download. This availability of comprehensive data was the rationale behind selecting heart failure for further analysis in our study. Our study leveraged two publicly accessible datasets: TCGA-BLCA (bladder cancer patients) and GSE116250 (patients with heart failure). The mRNA expression profiles, alongside other pertinent clinical information and prognostic outcomes, for the TCGA-BLCA dataset were retrieved from the GDC (Genomic Data Commons) website. Meanwhile, the GSE116250 dataset was sourced from the GEO (Gene Expression Omnibus) website. Additionally, two serum protein datasets from related studies were also incorporated into our subsequent analysis.

### Cross – WGCNA (Weighted Gene Co-expression Network Analysis) was used to identified the comorbidity genes’ modulus between bladder cancer and heart failure disease

The “WGCNA” (Weighted Gene Co-expression Network Analysis) package was utilized to identify comorbid genes between bladder cancer and heart failure [8]. Initially, the top 5000 variant coding mRNAs between cancerous and normal tissues from the TCGA-BLCA dataset were selected. Using the soft threshold provided by the “WGCNA” package, gene modules were determined. The module exhibiting the most significant difference between cancer and normal tissue was chosen for further analysis.

Initially, pearson’s correlation matrices and the average linkage method were both applied to analyze the relationships among all gene pairs. Subsequently, a weighted adjacency matrix was constructed employing a power function, specifically A_mn = |C_mn|^β, where C_mn denotes the Pearson’s correlation coefficient between Gene_m and Gene_n, and A_mn denotes the adjacency measure between Gene m and Gene n. The parameter β, serving as a soft-thresholding parameter, emphasized strong correlations between genes while penalizing weak ones. After selecting a power of 9, the adjacency matrix was transformed into a topological overlap matrix (TOM), which quantifies the network connectivity of each gene by summing its adjacencies with all other genes. Subsequently, the corresponding dissimilarity measure, defined as 1 minus TOM, was computed.

To organize genes with comparable expression profiles into distinct modules, average linkage hierarchical clustering was employed, utilizing the TOM-based dissimilarity measure. For the gene dendrogram, a minimum module size of 300 genes was established as a criterion. To further analyze the modules, the dissimilarity of module genes was calculated, a cut line for the module dendrogram was chosen, and some modules were merged.

Secondly, the module showing the most significant difference between heart failure patients and the control group in the GSE116250 dataset was selected using the aforementioned methodology.

Thirdly, a comparison was made between the two most distinct modules to identify comorbid genes between bladder cancer and heart failure. Additionally, these comorbid genes underwent Lasso regression ten times in the GSE116250 dataset to identify key proteins associated with heart failure.

Finally, a comparison of serum proteins between bladder cancer patients and heart failure patients was conducted to reveal potential comorbid serum proteins [9,10].

### Cell culture, plasmids, and transaction for bladder cancer cell line

A bladder cancer cell line, specifically the 5637 cell line, was acquired from the Authenticated Cell Culture Compilation of China. These cell lines were handled in accordance with the guidelines provided by Authenticated Cell Culture Compilation of China. For the purpose of transaction analysis, three siRNAs targeting the CAPG gene were obtained from General Biology Company situated in China. The efficacy of cell transaction was assessed using Quantitative PCR. Among the tested siRNAs, the one that demonstrated the lowest CAPG expression was selected for further functional studies. Additionally, over-expression plasmids for SFRP1 were also sourced from General Biology Company. Once again, Quantitative PCR was utilized to determine the effectiveness of SFRP1 over-expression.

### CCK-8 and Transwell assays

The CCK-8 assay was utilized to assess the proliferation of bladder carcinoma cells via a ECC Kit-8 (Enhanced Cell Counting Kit-8, Beyotime company, product id: C0037), Adhering strictly to the manufacturer’s recommended protocols. Utilizing Transwell assays, we assessed the migratory and invasive potential of bladder carcinoma cell lines.

### ELISA

The Human CAPG Elisa kit, sourced from EIAab company with the product ID E14072h, was utilized to quantify CAPG levels within cell line samples. The entire process adhered strictly to the manufacturer’s guidelines, ensuring accuracy and consistency. The plasma protein concentrations were expressed in terms of pg/ml for standardized measurement.

### Cell culture for Myocardial cell line

A Myocardial cell line (H9C2 cell line) were obtained from the Authenticated Cell Culture Compilation of China. The cell lines were treated following the guidelines of the Authenticated Cell Culture Compilation of China. CAPG (ZHI company, product id:ZI-21608) were using, in order to imitate the relationship between the serum protein of CAPG and Myocardial cell line.

Mitochondrial membrane potential in H9C2 cells was assessed using the TMRE (Tetramethylrhodamine, ethyl ester) staining method. Briefly, cells were seeded in 24-well plates and treated with different concentrations of recombinant CAPG protein. After 24 hours, cells were stained with TMRE staining solution and incubated at 37°C for 15 minutes. The mitochondrial membrane potential was then measured using a fluorescence microscope, with higher fluorescence intensity indicating higher membrane potential.

### The cell proliferation, the collagen I protein level, and mitochondrial membrane potential of myocardial cell were analyzed

Adhering strictly to the manufacturer’s recommended protocols, we employed the CCK-8 assay to evaluate the proliferation of myocardial cells using the ECC Kit-8 (Enhanced Cell Counting Kit-8) from Beyotime company (product ID: C0037). The Westbolt (WB) was used for the test of the collagen I protein level, with the collagen I protein antibody from proteintech company, product id: 67288–1-Ig. Using the myocardial cell, mitochondria were isolated using the myocardial mitochondria isolation kit (Beyotime company, product id: C2001S) and were used to detect the mitochondrial membrane potential.

Statistical analyses were performed using R (version 4.1.2). Descriptive statistics included mean, standard deviation, and median. Comparisons between two groups were made using t-tests or Mann-Whitney U tests, while multiple group comparisons used ANOVA or Kruskal-Wallis tests. Correlation analyses were conducted using Pearson’s or Spearman’s correlation coefficients. Survival analysis was performed using the Kaplan-Meier method and Cox proportional hazards model to assess survival differences and adjust for confounding factors. The significance level was set at 0.05, with multiple comparisons corrected to control the false discovery rate (FDR).

Ethical review and approval were waived following the local legislation and institutional requirements. Written informed consent for participation was not required for this study in accordance with national legislation and institutional requirements.

## Results

### The risk of CVD death gets significantly increased on the bladder cancer patients

A total of 313,743 diagnosed bladder cancer cases were identified in the SEER database (S1 Table). The exclusion criteria were as follows: (1) follow-up duration of less than 1 month or unknown; (2) unspecified variables, such as unknown clinical characteristics, surgery information, race, or pathological characteristics; (3) pathological characteristics falling under the 8th edition of the AJCC prognostic staging system. After applying these criteria, 140,760 eligible patients were included in the analysis, and their clinical information is presented in S2 Table. Following various medical treatments (surgery: 95%, chemotherapy: 21%, radiotherapy: 7%), 65,184 (46%) patients survived and continued to battle bladder cancer. The primary causes of death were bladder cancer (33,174), cardiovascular disease (CVD) (14,826), and other causes (27,576). Apart from the primary cancer, CVD emerged as the leading cause of death among bladder cancer patients. The population characteristics associated with these three causes of death are also presented in S3 Table.

To assess whether bladder cancer patients had an elevated risk of CVD-related death, the concept of standardized mortality ratio (SMR) was employed. The SMR was calculated by dividing the observed deaths by the expected deaths. The results confirmed our hypothesis, revealing that bladder cancer patients had an increased overall risk of CVD (+18%). This included diseases of the heart (+17%), hypertension without heart disease (+2%), cerebrovascular diseases (+6%), atherosclerosis (+16%), aortic aneurysm and dissection (+14%), and other diseases of the arteries, arterioles, and capillaries (+19%) (Table 1).

**Table 1:** The bladder cancer patients had worse CVD death risk.

Death of heart disease	SMR	Low 95% CI	High 95% CI
Total	1.18	1.14	1.2
Diseases of the heart	1.17	1.15	1.19
Hypertension without heart disease	1.02	1.01	1.05
Cerebrovascular diseases	1.06	1.02	1.11
atherosclerosis	1.16	1.11	1.19
Aortic aneurysm and dissection	1.14	1.10	1.17
Other diseases of the arteries, arterioles, and capillaries	1.19	1.02	1.38

Bladder cancer patients with more aggressive pathological features exhibited an elevated risk of cardiovascular disease (CVD) (Table 2). To investigate the association between bladder cancer pathology and CVD risk, we conducted a Cox regression analysis. The results demonstrated a statistically significant correlation. Specifically, bladder cancer progression was associated with an increased CVD risk, as indicated by the T stage (hazard ratio [HR], T2: 1.77, T3: 1.26, T4: 2.41; all P values < 0.001), N stage (HR, N1: 1.33, N2: 1.64; P values < 0.01), and M stage (HR, M1: 4.54; P value < 0.001). Notably, patients who underwent surgery for bladder cancer experienced a reduced CVD risk (HR, surgery: 0.77; P value < 0.001). Furthermore, surprisingly, we found that chemotherapy conferred benefits not only in inhibiting tumor progression but also in mitigating CVD risk among bladder cancer patients (HR, chemotherapy: 0.75; P value < 0.001). Traditionally, chemotherapy has been associated with cardiotoxicity and increased CVD risk. However, our findings suggest that the beneficial effects of chemotherapy on the heart in bladder cancer patients outweigh its toxic effects.

**Table 2:** Death of heart disease in the bladder cancer patients.

Characteristic	Death of Heart Disease		
	HR	95% CI	p-value
T2 vs T1	1.77	1.69, 1.86	<0.001
T3 vs T1	1.26	1.13, 1.41	<0.001
T4 vs T1	2.41	2.16, 2.70	<0.001
N1 vs N0	1.33	1.11, 1.59	0.002
N2 vs N0	1.64	1.33, 2.02	<0.001
M1 vs M0	4.54	3.89, 5.29	<0.001
Surgery: Yes vs No	0.77	0.71, 0.83	<0.001
Chemotherapy:Yes vs No/Unknown	0.75	0.71, 0.78	<0.001

Bladder cancer and heart disease affect two distinct organs in the human body, yet they share a common pathway: the blood circulation. We hypothesize that bladder cancer may secrete specific proteins into the bloodstream, potentially increasing the risk of cardiovascular disease (CVD). This suggests a possible link between the two conditions, mediated by the circulatory system.

### Cross-WGCNA analysis and Lasso regression found two key serum proteins: SFRP1, CAPG

**Fig 1:**
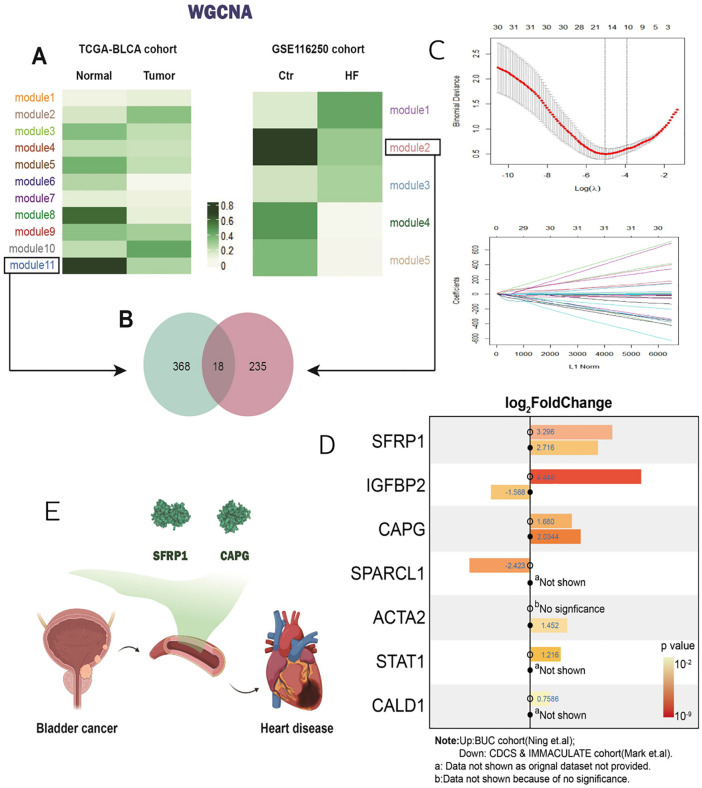
Cross-WGCNA analysis and Lasso regression identified two key serum proteins: SFRP1 and CAPG. A: Cross-WGCNA analysis results. Co-expression modules of genes were compared between bladder cancer patients and healthy individuals to identify gene modules related to cardiovascular disease. B: Interaction map of the two gene modules. The potential associations between the comorbidity gene modules of bladder cancer and cardiovascular disease are illustrated. C: Lasso regression analysis results. After multiple rounds of Lasso regression, seven genes, including SFRP1 and CAPG, were found to have significant differences in expression between the two diseases. D: Serum protein analysis results. The expression levels of serum proteins, including SFRP1 and CAPG, were compared among bladder cancer patients, heart failure patients, and healthy controls. E: Schematic diagram of the potential mechanism. The hypothetical mechanism by which bladder cancer may increase the risk of cardiovascular disease by regulating serum proteins such as SFRP1 and CAPG is depicted.

In the TCGA-BLCA dataset, 11 co-expression modules were identified through clustering analysis. Among these, module 11 exhibited the most significant difference in characteristics between cancer patients and healthy individuals, with a soft threshold of 0.38. Similarly, in the GSE116250 dataset, 5 co-expression modules were clustered, and module 2 displayed the most pronounced distinction for patients with heart failure, also using a soft threshold of 0.38. The intersection of these two modules revealed 18 comorbidity genes (Fig 1A-1B).

After performing Lasso regression ten times on the GSE116250 dataset, only 7 out of the 18 genes remained significant, namely SFRP1, SPARCL1, CAPG, IGFBP2, ACTA2, STAT1, and CALD1 (Fig 1C). Serum proteins from bladder cancer patients and heart failure patients were selected for further analysis in comparison to healthy controls.

The results indicated that three key serum proteins—IGFBP2, SFRP1, and CAPG—had significantly different expression levels in the serum of patients with both diseases (all P values < 0.05). These proteins may play a crucial role in the increased risk of cardiovascular disease (CVD) associated with bladder cancer. Given that IGFBP2 showed contrasting trends in the two disease types, SFRP1 and CAPG were selected for subsequent in molecular experiments to validate the hypothesis that bladder cancer might influence CVD death risk by regulating these serum proteins (Fig 1D-1E).

### Regulation the mRNA level of two key proteins had a significant impact on the bladder cancer’s proliferation, migratory and invasive capabilities

**Fig 2:**
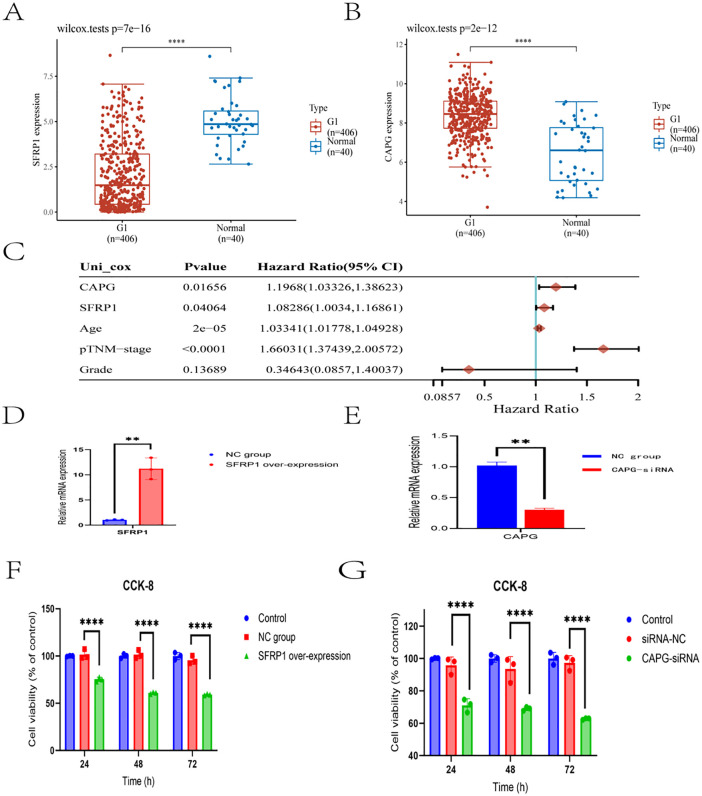
Regulation of SFRP1 and CAPG levels significantly impacts the proliferation of bladder cancer cells. A: Expression levels of SFRP1 in the TCGA-BLCA dataset. SFRP1 was significantly downregulated in cancer samples compared to normal samples (P < 0.0001). B: Expression levels of CAPG in the TCGA-BLCA dataset. CAPG was significantly upregulated in cancer samples compared to normal samples (P < 0.0001). C: Cox univariate analysis results. Both SFRP1 and CAPG were confirmed to have prognostic value in bladder cancer (P < 0.05). D: Overexpression of SFRP1 in cell transaction. Quantitative PCR analysis confirmed that SFRP1 mRNA levels were significantly higher in the overexpressing cell line than in the negative control (P < 0.01). E: CAPG siRNA interference in cell transaction. Quantitative PCR analysis confirmed that CAPG mRNA levels were significantly lower in the siRNA-interfered cell line than in the negative control (P < 0.01). F: CCK-8 analysis of SFRP1-overexpressing cell lines. Results showed that increased SFRP1 expression significantly inhibited the proliferation of bladder cancer cells (P < 0.01). G: CCK-8 analysis of CAPG siRNA-interfered cell lines. Results showed that decreased CAPG expression significantly inhibited the proliferation of bladder cancer cells (P < 0.01).

Firstly, the expression levels of two specific genes, SFRP1 and CAPG, were evaluated in the TCGA-BLCA dataset, which comprised of 406 cancer samples versus 40 normal samples. The analysis revealed significant differences in their expression patterns. Specifically, CAPG was found to be up-regulated in the cancer samples (P value < 0.0001), while SFRP1 exhibited down-regulation (P value < 0.0001, Fig 2A-2B). Additionally, Cox univariate analysis confirmed the prognostic value of both SFRP1 and CAPG in bladder cancer (P < 0.05, Fig 2C).

Secondly, to further investigate the roles of these genes, siRNAs and over-expression plasmids were utilized to modulate the expression of SFRP1 and CAPG in a bladder cancer cell line. Following cell transaction, cell lines with down-regulated CAPG and up-regulated SFRP1 were established. Quantitative PCR analysis confirmed significant differences in the expression levels of these two mRNAs compared to the negative control cell line (P value < 0.01, Fig 2D-2E).

**Fig 3:**
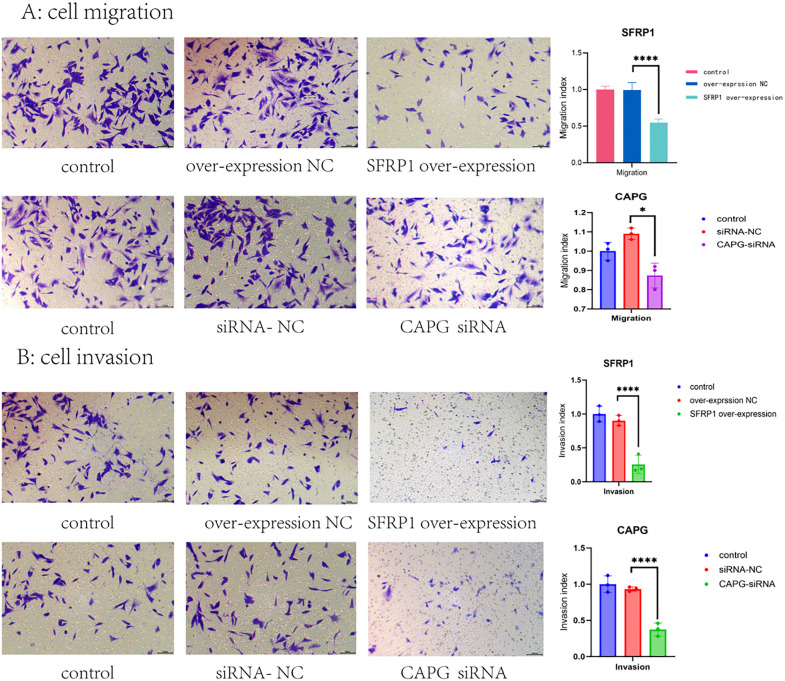
Regulation SFRP1 and CAPG level had a significant impact on the bladder cancer’s migratory and invasive capabilities. A: Analysis of migratory capabilities. Transwell assays showed that reduced CAPG levels or increased SFRP1 levels significantly inhibited the migration of bladder cancer cells (P < 0.001). B: Analysis of invasive capabilities. Transwell assays showed that reduced CAPG levels or increased SFRP1 levels significantly inhibited the invasion of bladder cancer cells (P < 0.001).

Thirdly, the functional impact of these altered gene expressions was assessed using CCK-8 and Transwell assays. These assays evaluated the proliferation, migration, and invasion capabilities of the modified bladder cancer cell lines. The results demonstrated that: 1. In the CCK-8 assay, a decrease in CAPG expression or an increase in SFRP1 expression significantly inhibited the proliferation of the bladder cancer cell line (P value < 0.01, Fig 2F-2G). 2.Similarly, in the Transwell assay, a reduction in CAPG levels or an elevation in SFRP1 levels led to a marked suppression of the migration and invasion abilities of the bladder cancer cells (P value < 0.001 for all comparisons, Fig 3A-3B).

### CAPG might be the key serum protein that the bladder cancer impacted the heart

**Fig 4:**
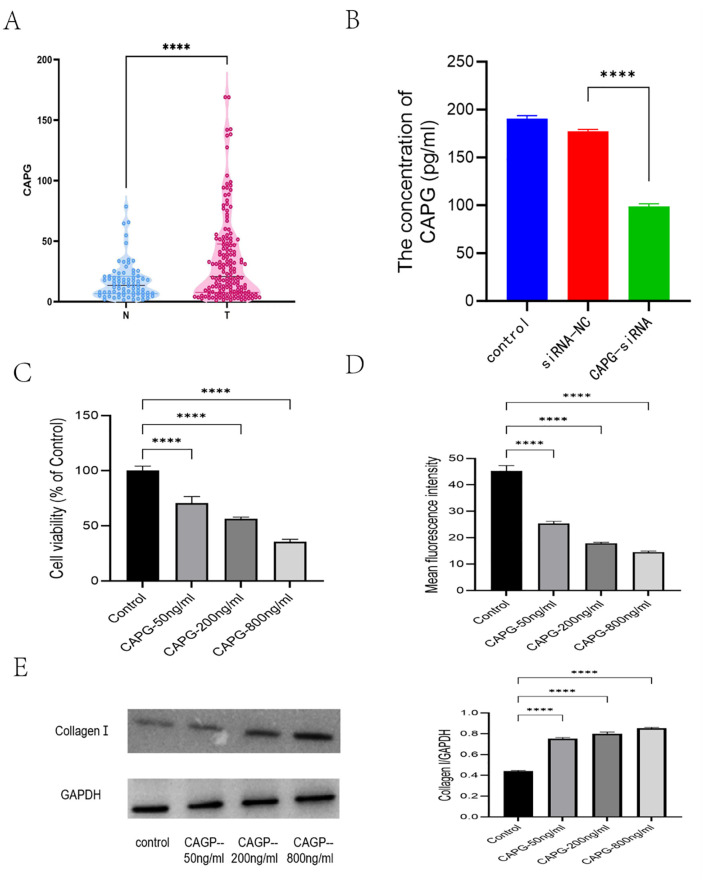
CAPG protein regulates myocardial function. A: Serum levels of CAPG in bladder cancer patients. Compared to healthy individuals, significantly higher levels of CAPG protein were found in the serum of bladder cancer patients (P < 0.001). B: ELISA analysis of CAPG protein in cell media. Results showed that the level of CAPG protein in the culture medium was significantly reduced in the CAPG siRNA-interfered cell line (P < 0.001). C: Effects of different CAPG protein concentrations on myocardial cell proliferation. CCK-8 assays indicated that higher concentrations of CAPG protein inhibited the proliferation of myocardial cells. D: Effects of different CAPG protein concentrations on mitochondrial membrane potential in myocardial cells. Results showed that increased CAPG protein levels led to a decrease in mitochondrial membrane potential. E: Effects of different CAPG protein concentrations on collagen I protein expression in myocardial cells. Western blot analysis showed that higher CAPG protein levels increased collagen I protein expression in myocardial cells.

Compared to SFRP1, CAPG emerged as a potentially more significant serum protein influenced by bladder cancer in its impact on the heart. Analysis of clinical data from bladder cancer patients revealed significantly higher levels of both CAPG and SFRP1 serum proteins compared to healthy individuals (P < 0.001, Fig 4A). However, further examination of protein levels in cancer tissue versus normal tissue indicated up-regulation of CAPG and down-regulation of SFRP1. It is hypothesized that the elevated SFRP1 serum levels may originate from secretion by other organs.

Secondly, to investigate the correlation between mRNA expression in bladder cancer cell lines and protein secretion levels, Elisa assays were conducted to measure protein concentrations in cell media. As anticipated, the siRNA-CAPG cell line exhibited notably reduced levels of CAPG protein in the cell medium (P < 0.001, Fig 4B).

These findings suggest that bladder cancers characterized by increased CAPG mRNA levels have the capacity to regulate the secretion of CAPG protein. This regulation may underlie the observed elevation of CAPG serum protein levels in patients with bladder cancer, potentially implicating CAPG as a key factor in the disease’s impact on cardiac health.

### The proteins of CAPG regulate myocardial function in a concentration dependent manner, including the proliferation, and mitochondrial membrane potential

To investigate the relationship between serum CAPG protein and myocardial cell line behavior, a myocardial cell line (H9C2) and human recombinant CAPG protein were procured. Experiments were conducted using three different concentrations of human recombinant CAPG protein (50ng/ml, 200ng/ml, 800ng/ml). The results indicated that higher concentrations of CAPG protein suppressed the proliferation of myocardial cells (Fig 4C) and reduced mitochondrial membrane potential (Fig 4D). Furthermore, increased CAPG protein levels led to elevated collagen I protein expression (Fig 4E). In conclusion, elevated CAPG protein concentration appears detrimental to the myocardial cell line, potentially explaining how elevated serum CAPG levels associated with bladder cancer could influence cardiovascular disease (CVD) mortality risk.

### SB525334 could not only inhibit the CAPG but also increase the ability of Cisplatin to kill the bladder cancer cell

Based on the aforementioned experiment, upregulation of CAPG not only enhances the proliferative, migratory, and invasive capabilities of bladder cancer but also impairs myocardial cell function. Following a series of investigations, SB525334 was identified as a compound capable of inhibiting CAPG expression.

**Fig 5:**
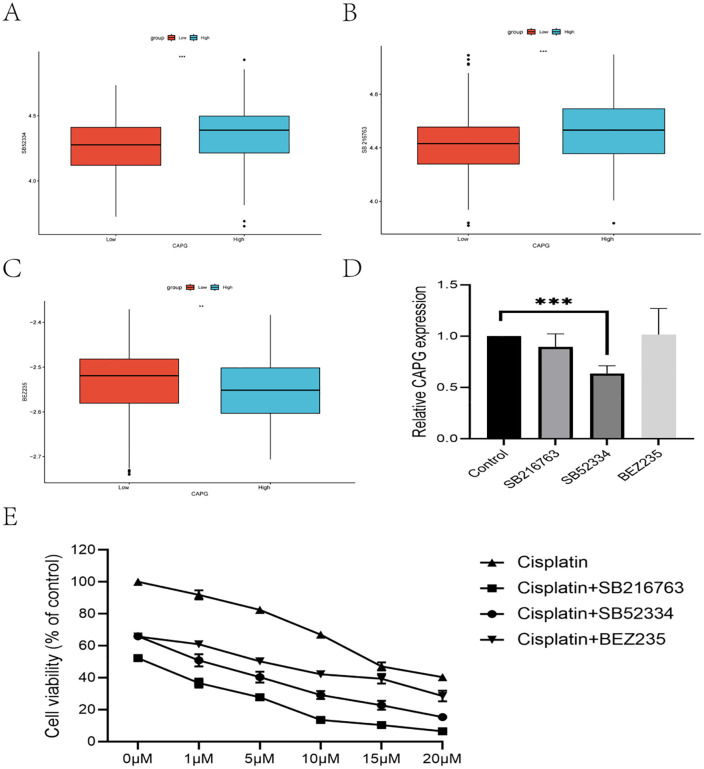
SB525334 not only inhibits CAPG expression but also enhances the cytotoxicity of cisplatin against bladder cancer cells. A: Drug sensitivity analysis of three potential drug candidates (SB525334, SB216763, and BEZ235) using the R package “pRRophetic” in the TCGA-BLCA dataset. B: Drug sensitivity analysis of SB216763. C: Drug sensitivity analysis of BEZ235. D: Effects of 20μM SB525334 on CAPG expression in bladder cancer cell lines. Quantitative PCR analysis showed that 20μM SB525334 significantly reduced CAPG expression in bladder cancer cell lines (P < 0.01). E: Drug sensitivity analysis of three drug candidates combined with cisplatin. CCK-8 assays showed that all three drug candidates, when combined with cisplatin, demonstrated satisfactory drug sensitivities. The IC50 values were 0.4471μM for SB216763 combined with cisplatin, 1.345μM for SB525334 combined with cisplatin, and 3.826μM for BEZ235 combined with cisplatin.

Firstly, to pinpoint more specific drug candidates targeting CAPG, we utilized the R package “pRRophetic” on the TCGA-BLCA datasets. This analysis revealed three potential drug candidates: SB525334, SB216763, and BEZ235 (Fig 5A-5C). These candidates exhibited varying degrees of drug sensitivity when BLCA patients from the TCGA-BLCA dataset evenly divided into two groups: one with high expression and the other with low expression (P < 0.01).

Secondly, to validate the inhibitory effects of these candidates on CAPG expression in bladder cancer cell lines, we obtained the following compounds: SB216763 (MEC company, product id: HY-12012), SB525334 (MEC company, product id: HY-12043), and BEZ235 (MEC company, product id: HY-50673). Our results demonstrated that SB525334 (20uM) significantly reduced CAPG expression in the bladder cancer cell line (P < 0.01, Fig 5D), while SB216763 (200uM) and BEZ235 (10uM) showed no significant difference, all three candidates’ concentrations were referred to relevant literature reports [11–13].

Thirdly, cisplatin is a first-line drug treatment for bladder cancer patients. We evaluated the drug sensitivity of three drug candidates (20μM SB216763, 200μM SB525334, or 10uM BEZ235) in combination with cisplatin (MEC company, product id: HY-17394) versus cisplatin alone in bladder cancer cell lines. Drug sensitivity was assessed using CCK-8 analysis and IC50 values. The results indicated that: 1.Cisplatin alone exhibited the lowest drug sensitivity with an IC50 value of 14.99μM. 2. All three drug candidates combined with cisplatin demonstrated satisfactory drug sensitivities. Specifically, the IC50 values were as follows: SB216763 combined with cisplatin had an IC50 value of 0.4471μM, SB525334 combined with cisplatin had an IC50 value of 1.345μM, and BEZ235 combined with cisplatin had an IC50 value of 3.826μM (Fig 5E).

## Discussion

Based on the concept that the human body is an organic whole, multiple diseases often exhibit characteristics of mutual influence and comorbidity [14]. The mechanisms by which two diseases interact are diverse and complex: 1) Many diseases share common pathophysiological pathways that serve as the basis for their mutual influence. For example, both cardiovascular disease and type 2 diabetes are associated with mechanisms such as insulin resistance, inflammation, and endothelial dysfunction [15]. These shared mechanisms may lead to changes in serum protein levels and establish a link between the two diseases. 2) Serum proteins can act as signaling molecules, transmitting information between cells and regulating physiological and pathological processes. In the context of mutual influence between two diseases, certain serum proteins may play a bridging role. For instance, one disease may affect the pathogenesis of another by altering the expression or function of serum proteins [16]. 3) The immune system also plays a significant role in the mutual influence of two diseases [17]. Certain serum proteins, such as inflammatory factors, can reflect the activation status of the immune system. When one disease leads to immune system activation, the released inflammatory factors may affect the pathogenesis of another disease. This immune-mediated interaction may be an important mechanism linking the two diseases [18].

The subject of this study is two types of diseases: bladder cancer and CVD. The bladder and the heart are two organs located at a considerable distance within the human body, and the most direct pathway connecting them is the blood circulatory system. Therefore, changes in key serum proteins became the first pathogenic mechanism considered by our team [19,20]. Finally, through a series of experiments, it was confirmed that bladder cancer can secrete CAPG protein, increasing the serum concentration of CAPG protein, and high concentrations of CAPG protein can inhibit the proliferation and membrane potential level of cardiomyocytes.

In this study, we demonstrated the clinical phenomenon and underlying mechanism of bladder cancer-induced CVD through four dimensions: 1. Based on clinical data, by analyzing the prognostic outcomes of bladder cancer in the SEER database, we clearly showed a statistical association between the clinical features of bladder cancer and CVD. 2. Using bioinformatics, we correlated bladder cancer with heart failure and identified three key serum proteins at both the tissue and serum levels. 3. Through molecular experiments, we further demonstrated that bladder cancer secretes CAPG protein, increasing its concentration in serum and inducing adverse outcomes in cardiomyocytes. 4. From a therapeutic perspective, we explored the use of a small molecule drug (SB525334) that can simultaneously control bladder cancer and reduce CAPG levels, protecting the heart.

Serum protein CAPG is a calcium-sensitive protein that functions in various cells, with particular emphasis on its association with macrophages [21]. Research on CAPG protein has mainly focused on its role in tumor development, where high expression of CAPG may induce proliferation and invasion in certain tumor cells, as confirmed in the bladder cancer studied in this research [22,23]. Secondly, the regulatory role of CAPG in immune response and inflammatory reactions has also received significant attention. There have been relatively few studies on the relationship between CAPG and heart disease [24]. However, this research provides sufficient evidence that upregulated extracellular CAPG concentration can significantly inhibit the proliferative capacity and membrane potential of cardiomyocytes.

The small molecule drug (SB525334) may be a potential therapeutic agent for controlling bladder cancer-induced CVD [25]. Firstly, the combination of SB525334 and cisplatin can significantly inhibit bladder cancer cells, helping to improve the progression of the primary bladder cancer disease. Secondly, SB525334 can suppress the expression level of CAPG in bladder cancer cells and also reduce the secretion level of CAPG from bladder cancer cells. This can reduce damage to cardiomyocytes, protect the heart, and ultimately reduce the risk of CVD. Lastly, the treatment regimen of SB525334 combined with cisplatin can achieve the desired therapeutic effect using a smaller dose of cisplatin, which undoubtedly provides the possibility of a low-dose chemotherapy regimen for existing chemotherapy protocols. The damaging effects of chemotherapy on cardiomyocytes are closely related to drug concentration. By reducing the dose of chemotherapy drugs, this regimen can reduce chemotherapy-related cardiotoxicity and have a protective effect on the heart.

In the future, our research will focus on further analyzing the role of CAPG protein in animal models and clinical patients, and exploring the potential functions of SB525334 in controlling bladder cancer, inhibiting CAPG levels, and protecting the heart. Based on our findings, we propose a potential mechanistic pathway linking bladder cancer and cardiomyopathy. Bladder cancer cells secrete high levels of CAPG protein, which enters the bloodstream and binds to receptors on cardiomyocytes. Elevated CAPG levels can inhibit the proliferation and mitochondrial membrane potential of cardiomyocytes, leading to impaired cardiac function and increased risk of cardiovascular diseases (CVD). Additionally, CAPG may activate downstream signaling pathways, such as the TGF-β signaling pathway, which is known to be involved in fibrosis and inflammation, further contributing to cardiomyopathy. Future studies will focus on elucidating the specific downstream targets of CAPG in cardiomyocytes and exploring potential therapeutic interventions targeting this pathway.

## Conclusion

Patients with bladder cancer have an increased risk of CVD. High expression of CAPG protein in bladder cancer can induce upregulation of serum CAPG protein levels and inhibit cardiomyocytes.

## Supporting information

S1 Table A total of 313,743 diagnosed bladder cancer cases were identified in the SEER database. (XLSX)

S2 Table 140,760 eligible patients were included in the analysis, and their clinical information is presented. (XLSX)

S3 Table The population characteristics of all death bladder cancer patients were presented. (XLSX)

S1 Fig The original picture of Collagen I WB blot. (TIF)

S2 Fig The original picture of GAPDH WB blot. (TIF)
