# Supplementary figures and images for: Bladder cancer-induced CVD mortality: Role of CAPG protein
Source: PLoS One. 2026 Feb 17;21(2):e0338101. doi: 10.1371/journal.pone.0338101 (PMC12912693; doi:10.1371/journal.pone.0338101)

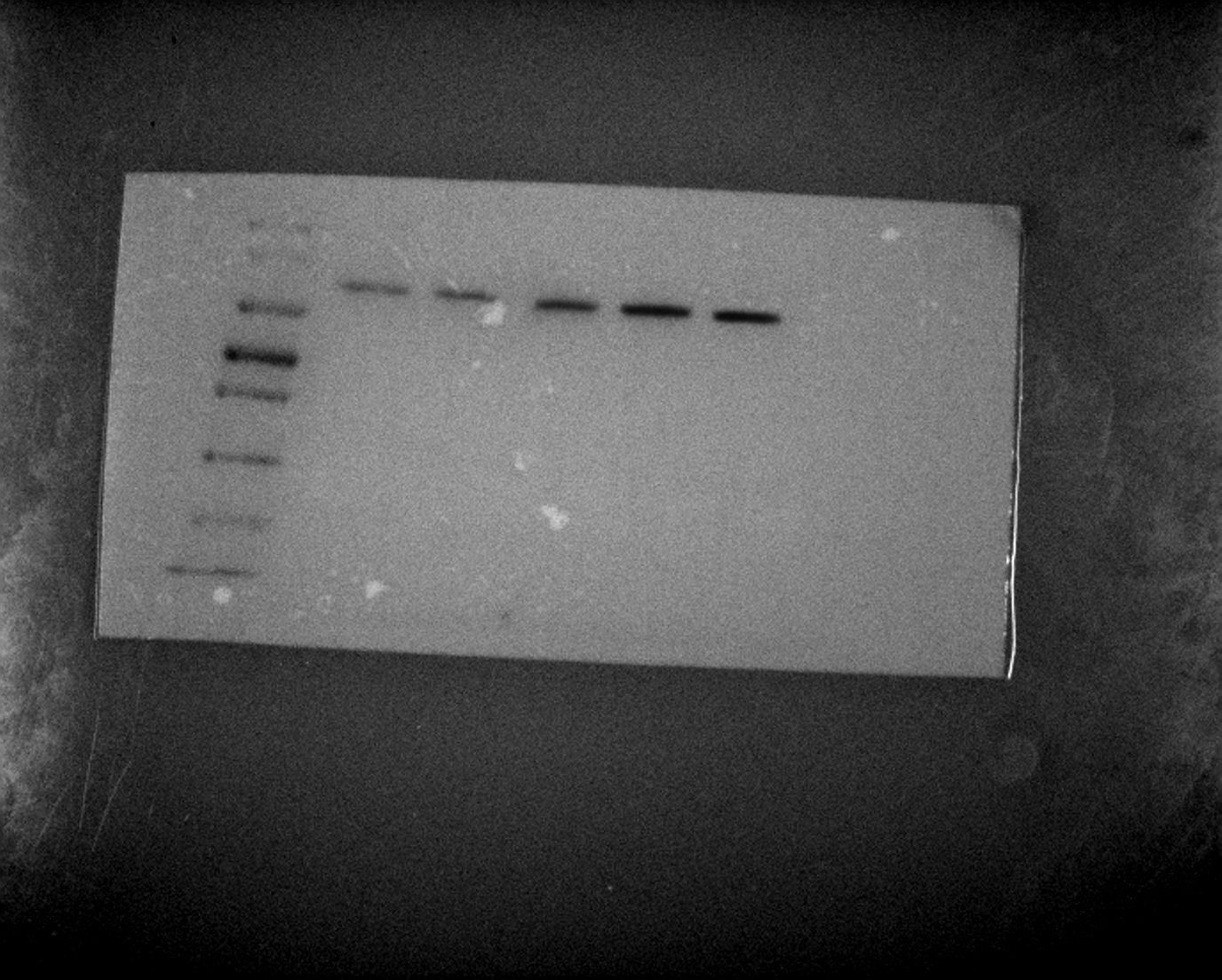

Supplement: S1 Fig — (TIF) [file pone.0338101.s004.Tif]

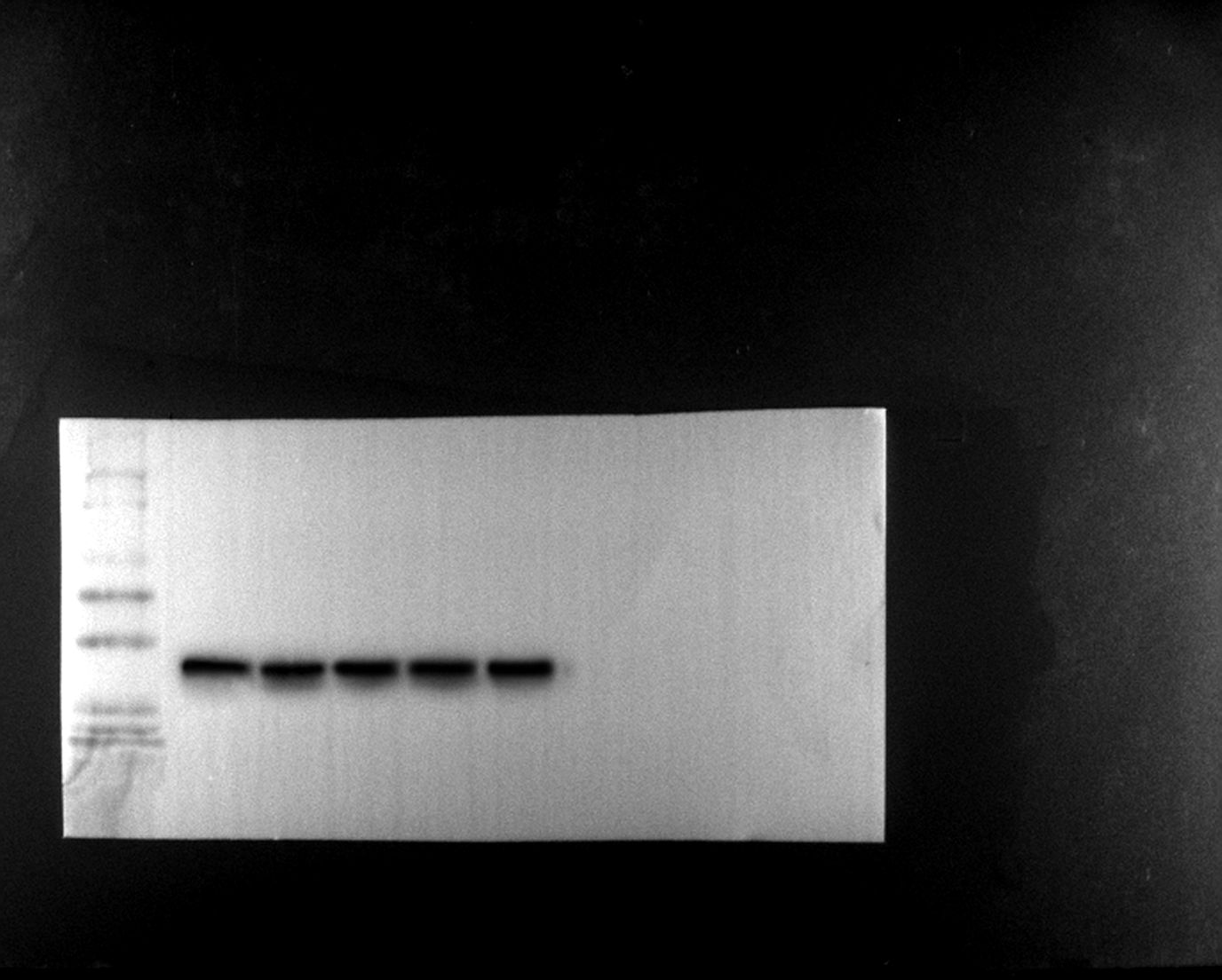

Supplement: S2 Fig — (TIF) [file pone.0338101.s005.tif]
